# Accelerating fragment-based library generation by coupling high-performance photoreactors with benchtop analysis

**DOI:** 10.3762/bjoc.16.87

**Published:** 2020-05-12

**Authors:** Quentin Lefebvre, Christophe Salomé, Thomas C Fessard

**Affiliations:** 1 SpiroChem AG, Rosental area, WRO-1047-3, Mattenstrasse 24, 4058 Basel, Switzerland

**Keywords:** benchtop analytics, fragment-based library, heterocyclic sp^2^–sp^3^ fragments, *N*-arylation, photoredox-nickel dual catalysis

## Abstract

Herein we report a workflow coupling photoredox-nickel dual-catalyzed *N*-arylation reactions to benchtop analysis for the efficient generation of fragment-based libraries. Technological advances in photoreactor design facilitated reliable and reproducible experimentation. Knowledge on the reactivity under previously reported reaction conditions of spirocyclic and strained heterocyclic building blocks, viewed as chemistry informers, could thus be rapidly accessed, identifying privileged or challenging scaffolds and paving the way for further exploration.

## Introduction

Heterocyclic sp^2^–sp^3^ fragments are sought-after in fragment-based libraries, as they offer improved pharmacokinetic properties over more classical, “flat” scaffolds [1]. Specifically, bicyclo- and spiroalkanes are sp^3^-rich (more 3D-like), conformationally-restricted and strained building blocks that are under-represented in screening collections. These are of high value to medicinal chemists to provide defined exit vectors and facilitate rational lead evolution [2–7]. Thus, we were interested in developing reliable synthetic methods and technologies to apply metal-catalyzed cross-coupling reactions to such scaffolds, which often exhibit different reactivity from unstrained substrates [8]. *N*-Arylation of spirocyclic amines would be a very efficient strategy for the modular synthesis of heterocyclic sp^2^–sp^3^ fragments, but their lack of stability to strongly basic conditions and heating might be an issue in palladium-catalyzed cross-coupling reactions [9]. Recently, milder cross-coupling methods using more abundant and affordable metals such as nickel, in combination with photochemistry or electrochemistry, significantly improved accessibility to *N*-arylated complex amines [10–12].

## Results and Discussion

**Scheme 1:**
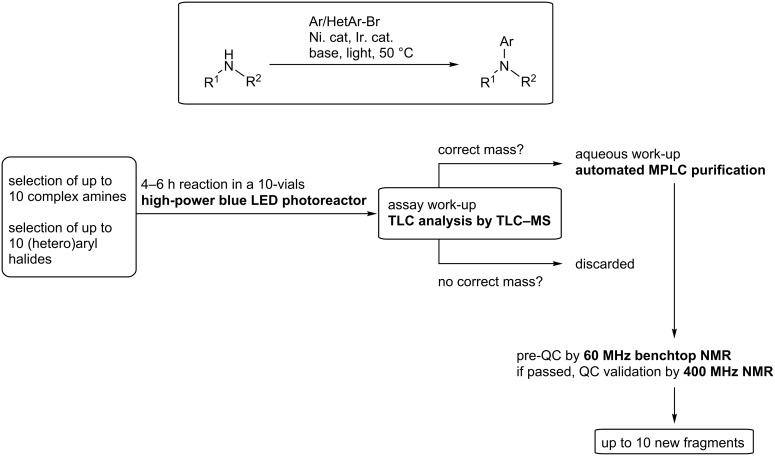
One-day workflow for fragment-based library generation. QC: Quality control. MPLC: Medium-pressure liquid chromatography.

As we initiated a high-throughput program of fragment-based library generation and after shortcomings using more classical cross-coupling conditions, we turned our attention to the photoredox-nickel dual-catalysis strategy recently reported by MacMillan and Buchwald [10]. The reported conditions use lower temperatures and a mild base. Inspired by work at Merck on chemical informers, we decided to exploit our library of spirocyclic amines and strained scaffolds to generate a focused list of potential products [13]. We wanted to perform parallel reactions on small scale to gain further knowledge on the reactivity of these building blocks for future applications. Reaction times had to be short, and analysis, work-up and purification had to be streamlined. To comply with these project specifications, and because of the relative high cost of starting amines, the reported conditions of dual-catalyzed *N*-arylation were slightly altered: a smaller excess of amine was used, and dimethyl sulfoxide (DMSO) was chosen as solvent instead of dimethylformamide (DMF) or dimethylacetamide (DMA) to improve work-up and purification. The workflow was devised to ensure maximal productivity, using parallel experimentation coupled to benchtop analysis tools, namely thin-layer chromatography–mass spectrometry (TLC–MS, purchased from https://www.advion.com/rsc-product-note/direct-mass-analysis-of-tlc-plates/) and low-field nuclear magnetic resonance (NMR, purchased from https://www.nanalysis.com/) analysis (Scheme 1).

**Figure 1:**
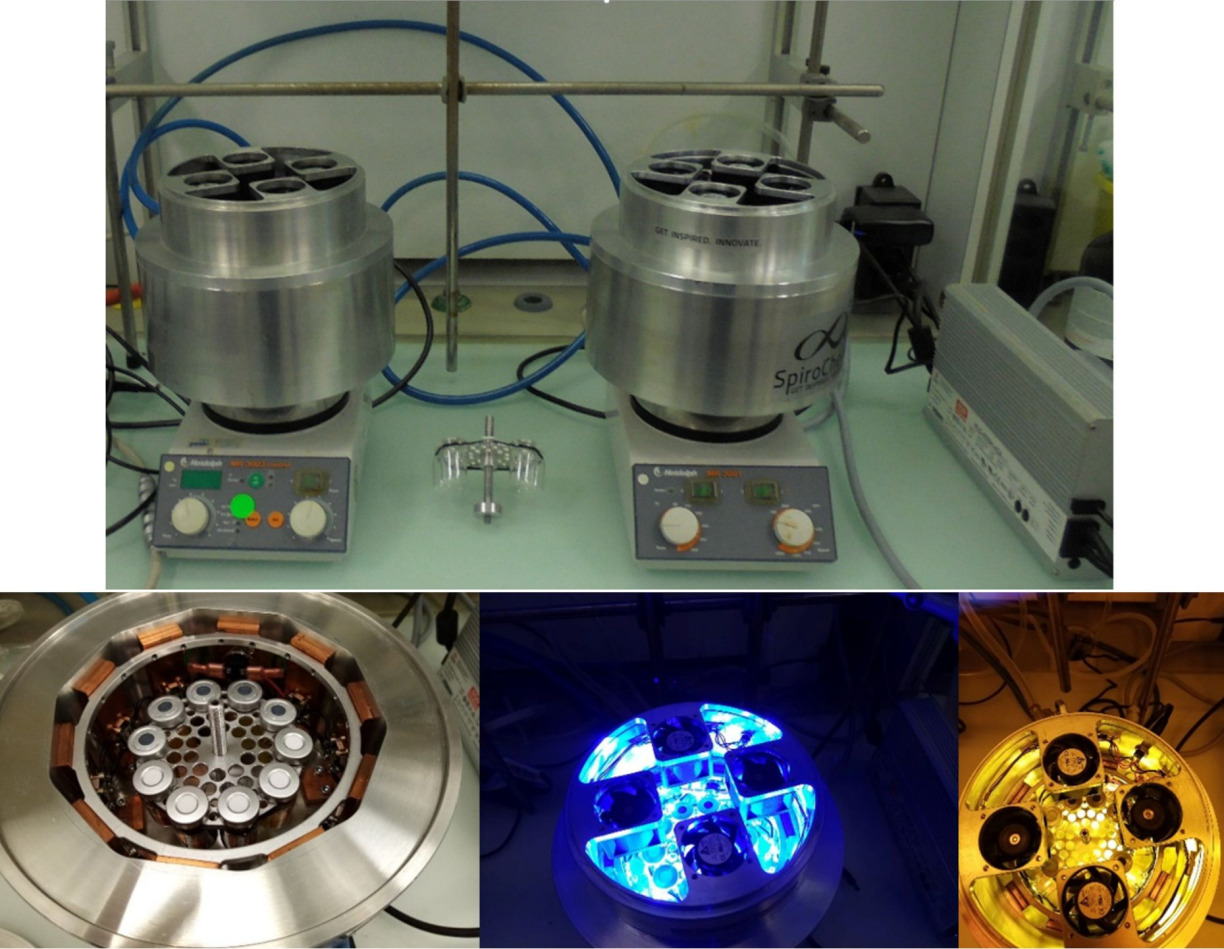
Top: high-power blue LED photoreactors. Bottom, from left to right: photoreactor OFF, ON, and ON through protective yellow filter.

To ensure reproducibility of the reactions, we used a custom-made, high intensity photoreactor which enables accurate control of irradiation and heat [14–15]. Indeed, homogeneous irradiation and temperature in screening decreases the risk of false negatives triggered by high variability of reaction conditions compared to a single vial reactor. Active water cooling protects the LEDs from overheating, while additional air cooling with bespoke fans prevents the vessels to heat over 50 °C. Each vessel is placed at equal distance of a 35 W high power blue LED (Figure 1).

**Figure 2:**
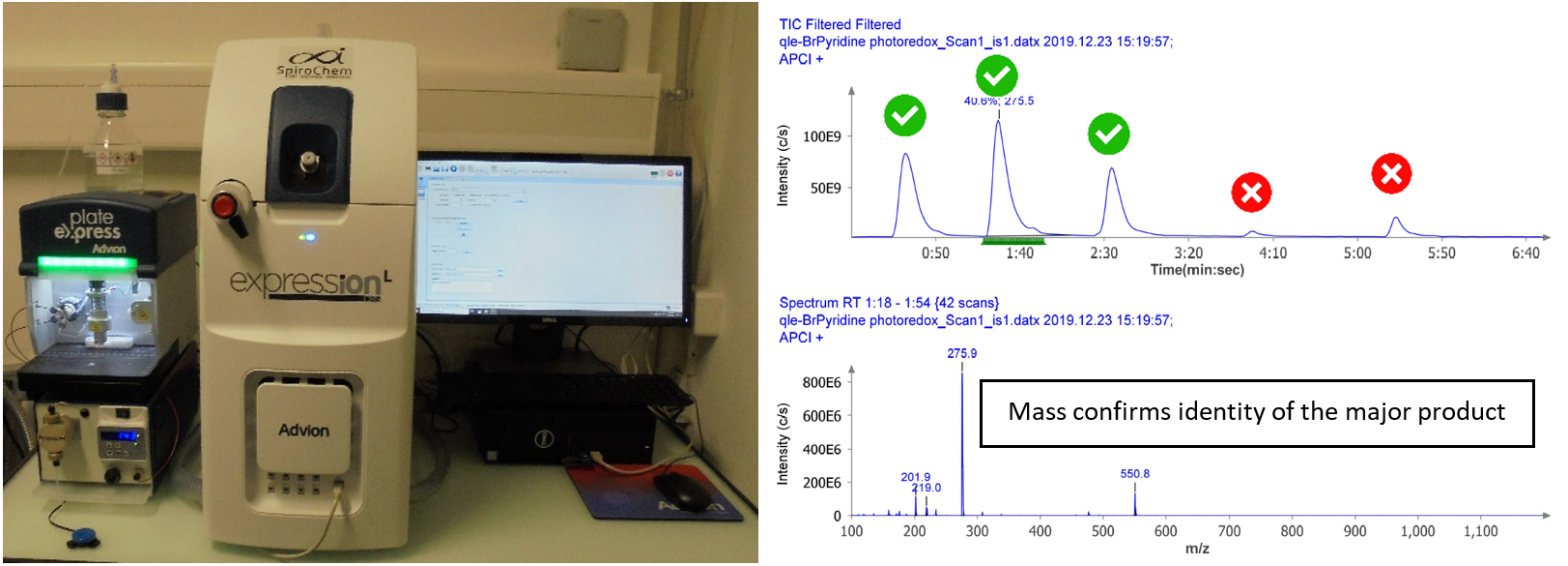
TLC–MS equipment: analysis of 5 reactions in 5 minutes.

Reaction monitoring by TLC–MS enabled rapid and precise assessment of the success of each parallel reaction. As seen in Figure 2, a single TLC plate reporting five different reactions could be analyzed in five minutes, and the identity of the major product confirmed by mass.

**Figure 3:**
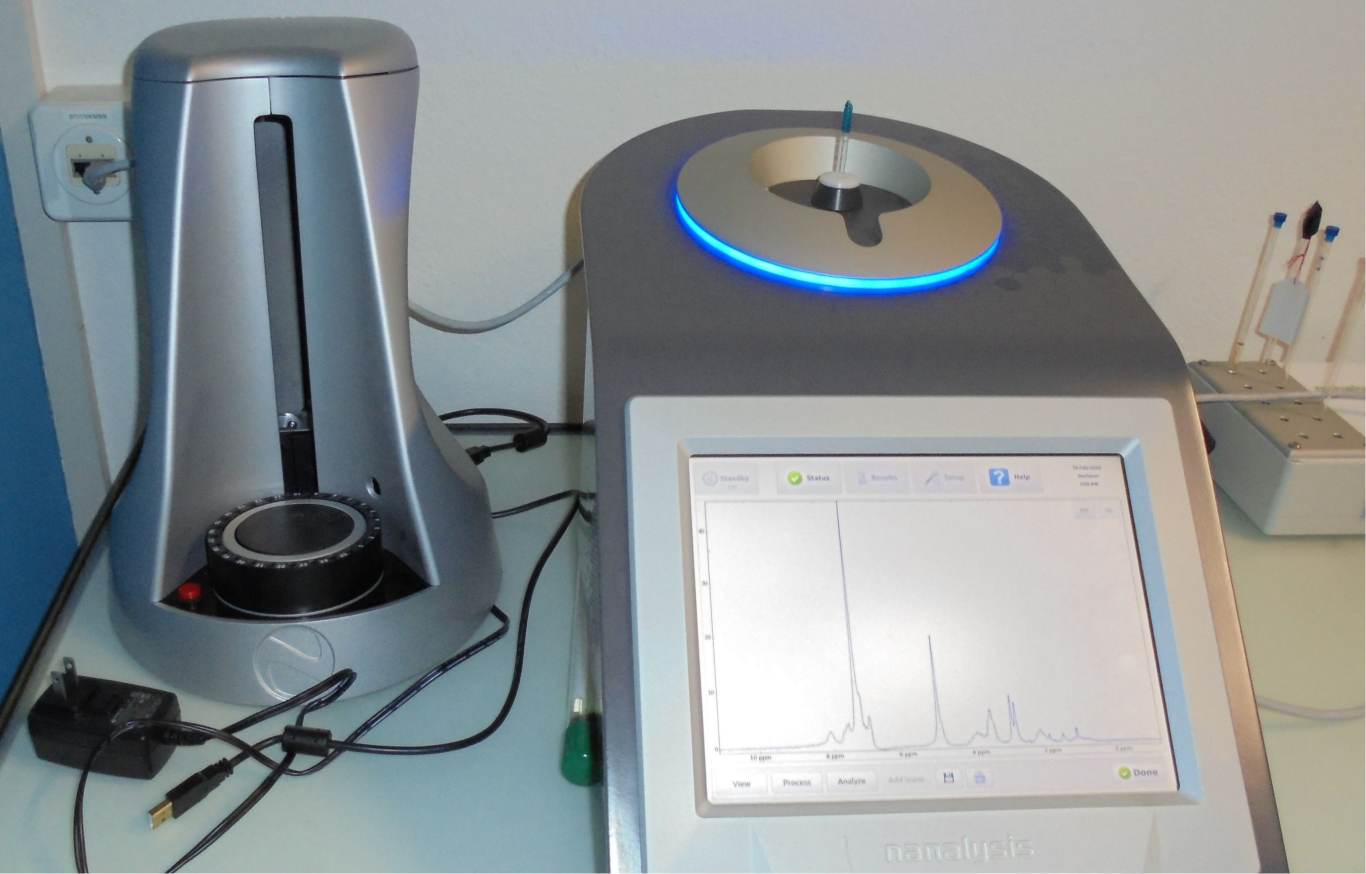
Pre-QC validation by 60 MHz benchtop NMR.

Finally, after work-up and automated purification, a preliminary quality control (QC) could be performed using a benchtop 60 MHz NMR machine (Figure 3). Structural information was obtained to corroborate mass spectrometry data, an important information when dealing with strained bicycles or spirocycles, which could be prone to skeletal rearrangement. If diagnostic peaks of solvent impurities coming from the column eluents were observed, the purified material was dried further. Thus, costly and time-consuming measurements using a high-field NMR machine could be avoided: Only the final QC was performed on a 400 MHz NMR machine.

**Scheme 2:**
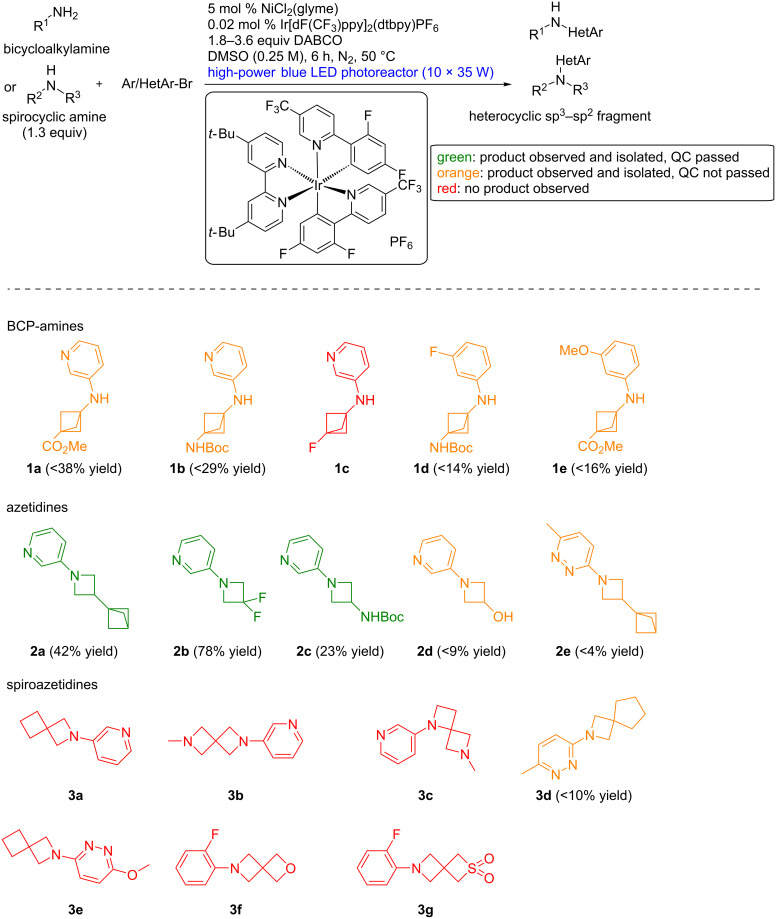
Scope of the fragment-based library generation: BCP-amines and azetidines. See Supporting Information File 1 for experimental details.

**Scheme 3:**
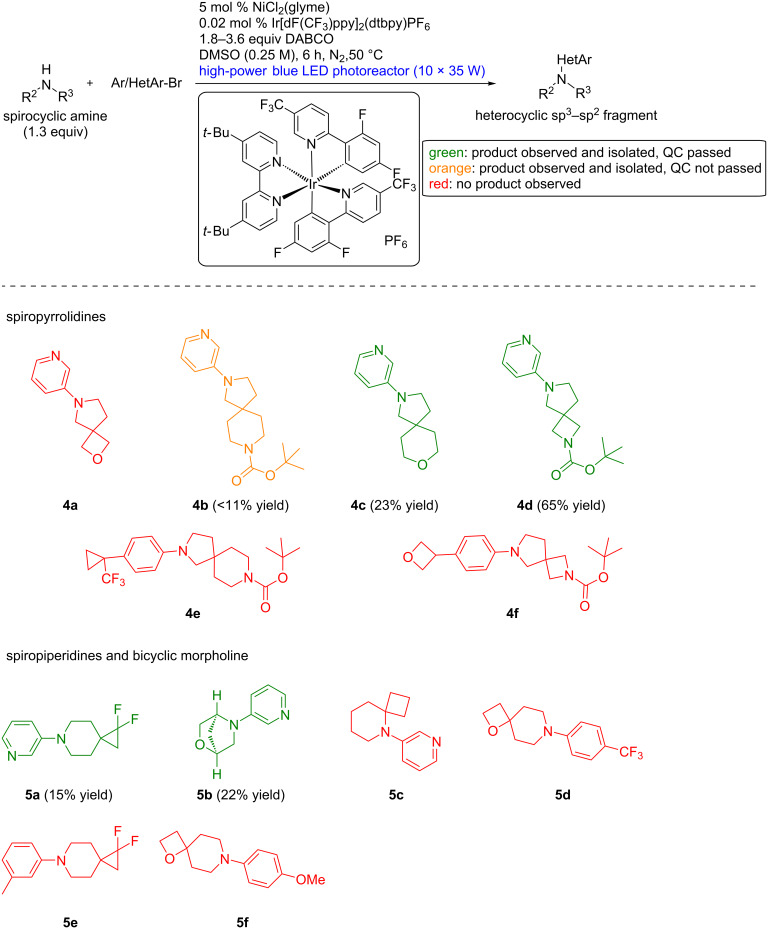
Scope of the fragment-based library generation: pyrrolidines, piperidines and morpholines. See Supporting Information File 1 for experimental details.

In 4 days of experimentation, 29 combinations of bicycloalkylamines or spirocyclic amines with (hetero)aryl bromides were probed using this workflow, and the results are depicted in Scheme 2 and Scheme 3. To provide attractive heterocyclic sp^2^–sp^3^ fragments, we mainly focused on coupling 3-bromopyridine to five different classes of complex amines: bicyclopentanamines, azetidines, spiroazetidines, spiropyrrolidines and spiropiperidines. Functional group tolerance was probed by using functionalized building blocks bearing esters, carbamates, alcohols, tertiary amines, ethers, sulfones and fluorine atoms (Scheme 2 and Scheme 3).

Out of the 15 reactions showing product formation by TLC–MS, 7 gave the product in sufficient purity after one round of purification, while 8 were contaminated with solvent impurities, or obtained in too low yields for appropriate NMR analysis. Fourteen did not give the expected product, often showing low consumption of the aryl bromide. This corresponds to a success rate of 24% for product purification and isolation, and 52% for product formation. Although our focused library is not as functional-group-dense as the chemistry informer library used by Merck, these results are in line with previous findings on C–N cross-coupling conditions. In these works, nickel-photoredox-catalyzed cross-couplings were the most successful with success rates up to 50% [10]. This was an improvement from non-photocatalyzed conditions, where success rates of 11–33% were observed for palladium-catalyzed cross-coupling reactions, and ranged from 6% to 50% for copper-catalyzed cross-coupling reactions [13].

Much information could be obtained from this screening. Electron-deficient aryl bromides led to better yields than neutral and electron-rich partners, as observed in previous reports on photochemically- or electrochemically-mediated nickel-catalyzed cross-couplings. Electron-deficient aniline products are less prone to oxidative decomposition. BCP-amines were viable coupling partners but gave the corresponding products **1a–e** in poor purities. Simple azetidines partook the reaction to give **2a–c** while azaspiro[3,3]heptane analogues were unreactive. The higher reactivity of azaspiro[3,4]octane example **3d** highlights the distinct reactivity of azaspiro[3,3]heptanes. On one hand, a rationalization of this observation could be traced back to the first step of the catalytic cycle. Reduction of the nickel(II) pre-catalyst to nickel(0) is believed to occur by β-hydride elimination on a sacrificial amount of amine [10]. The strained character of azaspiro[3,3]heptane might prevent this event. On the other hand, pyramidalization at nitrogen is much more important in azetidines [16–18]. This might lower the oxidation potential of the arylated product and promote oxidative decomposition. Several spirocyclic compounds with larger ring sizes could be coupled to 3-bromopyridine to give products **4c**, **4d, 5a** and **5b**, provided that steric hindrance was not too high (see **5c**). Surprisingly, oxetanes appear to be incompatible with the reaction conditions (see **4a**, **4f**, **5d** and **5f**), despite a successful report on their decarboxylative arylation under nickel-photoredox-catalyzed conditions [19]. As this report and MacMillan’s report use more basic, but less nucleophilic bases than DABCO, namely Barton’s base and MTBE, the issue might come from ring opening [20–21].

## Conclusion

In summary, we have developed a new workflow allowing to efficiently perform photoredox-nickel dual-catalyzed reactions in parallel for fragment-based library generation. We have screened 29 combinations of complex spirocyclic amines and (hetero)aryl halides to get an insight into the specific reactivity of these building blocks. The workflow combined a powerful photoreactor with precise and reproducible conditions control with benchtop analytical tools such as TLC–MS and low-field NMR. This enabled the identification of privileged spirocyclic scaffolds compatible with this chemistry, and challenging substrates requiring additional investigations. The knowledge obtained within this four-day experimentation period could be built-upon in-house and allowed the efficient synthesis of original heterocyclic sp^2^–sp^3^ fragments.

## Supporting Information

File 1 Experimental part.
