# Supplementary material for: Accelerating fragment-based library generation by coupling high-performance photoreactors with benchtop analysis
Source: Beilstein J Org Chem. 2020 May 12;16:982–8. doi: 10.3762/bjoc.16.87 (PMC7237803; doi:10.3762/bjoc.16.87)

BEILSTEIN JOURNAL OF ORGANIC CHEMISTRY

Supporting Information

for

Accelerating fragment-based library generation by coupling high-performance photoreactors with benchtop analysis

Quentin Lefebvre, Christophe Salomé and Thomas C. Fessard

Beilstein J. Org. Chem. **2020,** *16,* 982–988. doi:10.3762/bjoc.16.87

Experimental part

License and Terms: This is a supporting information file under the terms of the Creative Commons Attribution License (http://creativecommons.org/licenses/by/4.0). Please note that the reuse, redistribution and reproduction in particular requires that the authors and source are credited. The license is subject to the *Beilstein Journal of Organic Chemistry* terms and conditions: (https://www.beilstein-journals.org/bjoc)

Contents

General Information S2

Preparation and characterization of the products S3

General procedure S3

3-(3-(Bicyclo[1.1.1]pentan-1-yl)azetidin-1-yl)pyridine **2a** S5

3-(3,3-Difluoroazetidin-1-yl)pyridine **2b** S6

tert-Butyl (1-(pyridin-3-yl)azetidin-3-yl)carbamate **2c** S7

2-(Pyridin-3-yl)-8-oxa-2-azaspiro[4.5]decane **4c** S8

tert-Butyl 6-(pyridin-3-yl)-2,6-diazaspiro[3.4]octane-2-carboxylate **4d** S9

1,1-Difluoro-6-(pyridin-3-yl)-6-azaspiro[2.5]octane **5a** S10

(1S,4S)-5-(Pyridin-3-yl)-2-oxa-5-azabicyclo[2.2.1]heptane **5b** S11

General Information

All reactions were performed with oven-dried glassware and under an inert atmosphere (nitrogen) unless otherwise stated. All solvents were used as purchased unless otherwise stated. Commercial reagents were used as purchased without further purification. Organic solutions were concentrated under reduced pressure on a Büchi rotary evaporator. Thin-layer chromatography was carried out using Merck Kieselgel 60 F254 (230–400 mesh) fluorescent treated silica and were visualized under UV light (250 and 354 nm) and/or by staining with aqueous potassium permanganate solution. ^{1}H NMR spectra were recorded in deuterated solvents on Bruker spectrometer at 400 MHz or Nanalysis NMReady-60PRO spectrometer at 60 MHz, with residual protic solvent as the internal standard. ^{13}C NMR spectra were recorded in deuterated solvents on Bruker spectrometer at 100 MHz, with the central peak of the deuterated solvent as the internal standard. Chemical shifts (δ) are given in parts per million (ppm) and coupling constants (*J*) are given in Hertz (Hz) rounded to the nearest 0.1 Hz. The ^{1}H NMR spectra are reported as δ/ppm downfield from tetramethylsilane (multiplicity, number of protons, coupling constant *J*/Hz). The ^{13}C NMR spectra are reported as δ/ppm. TLC–MS data was obtained on Advion Expression CMS coupled with Plate Express TLC-plate Reader. Medium pressure liquid chromatography (MPLC) was performed on a Biotage Isolera Four with built-in UV-detector and fraction collector with Interchim silica gel columns.

High Intensity Photoreactors were custom designed and built in coordination with the mechanical workshop in the Department of Chemistry and Biosciences at ETH Zürich having blue LEDs, equally spaced in a circle design, powered by a 10.3 A power supply, emitting 350 W of light. The LEDs were water cooled and further cooled by built-in fans to maintain an ambient temperature.[1] See this reference for UV–vis emission spectrum.

The photoreactor is circular, with 10 high-power LEDs equally distributed along the perimeter. The vial holder can contain 10 vials also equally distributed. This way, each vial can be positioned in front of each LED so that each reaction receives the same amount of light, mostly from the LED directly in front of it. The distance and the angle between the LED and the vial are the same for all 10 vials, making sure that irradiation is consistent within one series of screening. To further validate reproducibility, each series of screening contained a common reference reaction, the yield of which was found constant across different series.

All reactions were performed at the same temperature, controlled by a combination of water and air cooling. The use of the thermometre probe of the stirring plate showed that the inner temperature of a DMSO solution in the same vial used for the reactions reached 40 °C within 2 minutes. This value stabilized at 50 °C after 10 minutes and stayed at this value for the duration of the experiment (6 hours). Thus, preheating of the photoreactor was not deemed necessary.

[1] B. J. Jelier et al, *Angew. Chem. Int. Ed.* **2018**, *57*, 13784.

Preparation and characterization of the products

General procedure

In a 5 mL crimp neck vial, Ir[dF(CF_3)ppy]$_2$(dtbpy)PF_6 (0.0002 equiv, 0.15 µmol, 0.2 mg), $NiCl_2$(glyme) (0.05 equiv, 38 µmol, 8.2 mg), amine as a free base, hydrochloride salt or hemioxalate salt (1.3 equiv, 0.98 mmol) DABCO (1.8–3.6 equiv depending on the nature of the amine, 1.35–2.70 mmol, 0.150–0.300 g) were placed sequentially. Dry DMSO (3 mL) was added, followed by the aryl bromide (1.0 equiv, 0.75 mmol), and the mixture was degassed by bubbling nitrogen gas through the reaction mixture for one minute. The vial was sealed with a crimp cap, placed on the photoreactor holder and irradiated with high-intensity blue LEDs for 6 h. An aliquot was taken and partitioned between water and ethyl acetate. The crude was analysed by TLC and the major spots were analysed by TLC–MS.

If the product was observed by TLC–MS, the crude was diluted with ethyl acetate (25 mL) and washed three times with water (3 × 50 mL) and with brine. The organic layers were combined and dried over sodium sulphate, filtered, evaporated and purified by automated column chromatography on silica gel. The collected fractions containing the product were evaporated and the residue dried until the 60 MHz NMR spectrum did not show any solvent to the limit of detection.

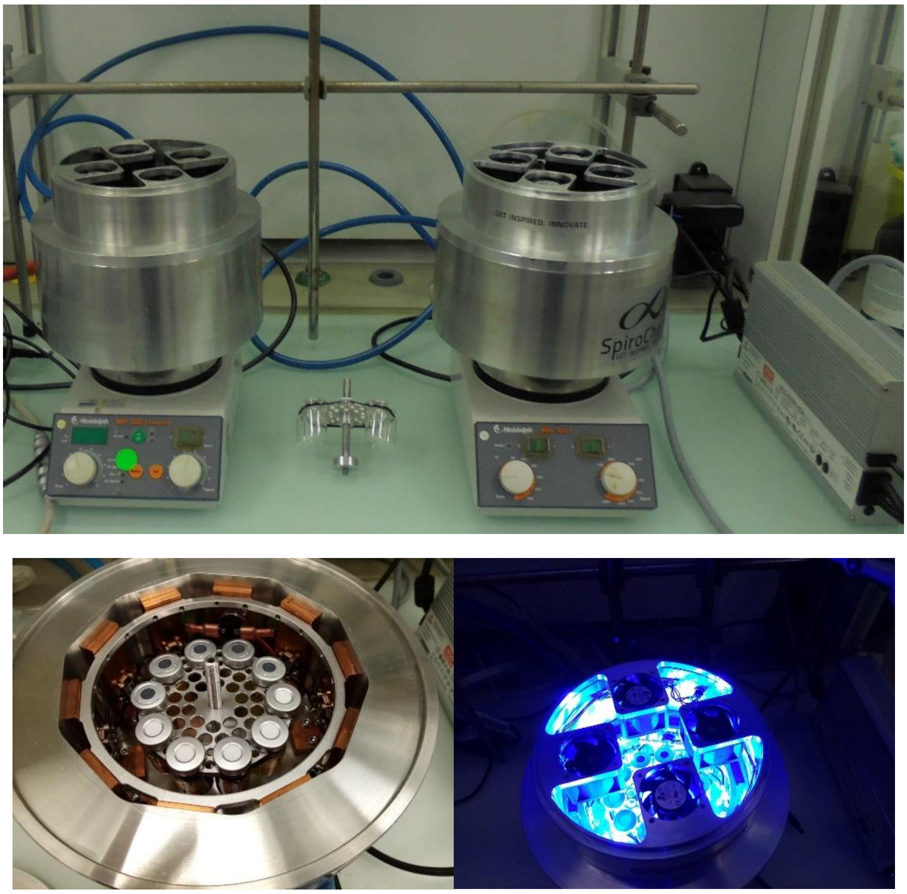

Figure S1: High-intensity photoreactors used in this study.

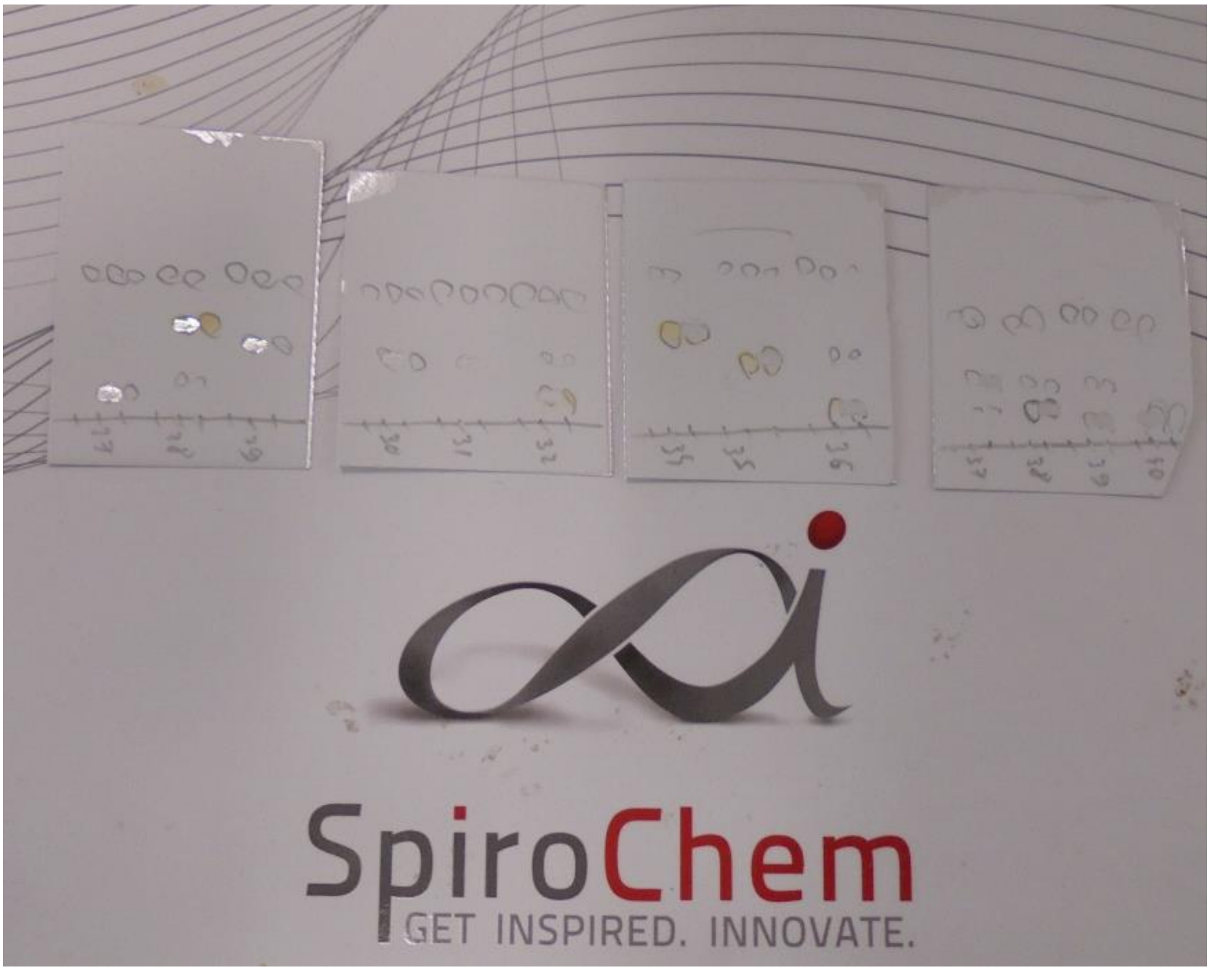

Figure S2: Array of 13 parallel reactions analyzed by TLC–MS.

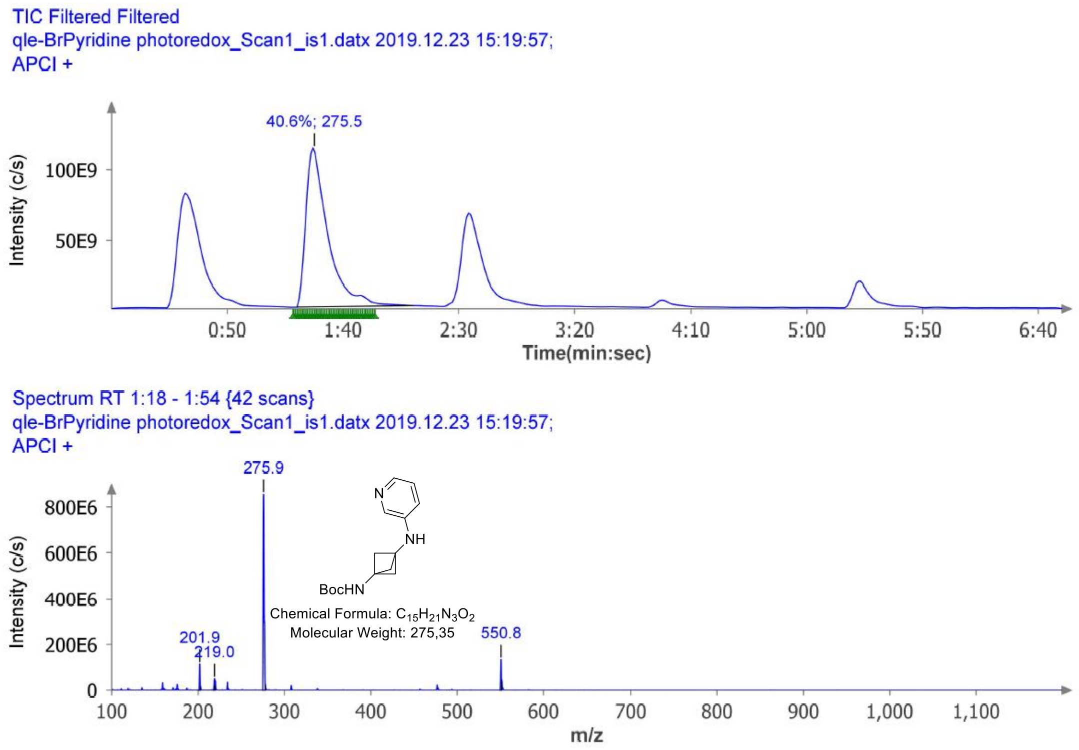

Figure S3: Example of a set of five reactions analysed in five minutes by TLC–MS.

3-(3-(Bicyclo[1.1.1]pentan-1-yl)azetidin-1-yl)pyridine (**2a**)

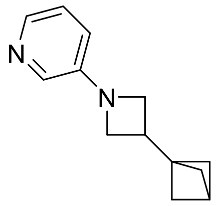

Yellow solid, 63 mg, 42% yield.

^{1}H NMR (400 MHz, $CDCl_3$): δ 7.99 (bs, 1H), 7.80 (bs, 1H), 7.03 (bs, 1H), 6.66 (bs, 1H), 3.86 (bs, 2H), 3.56 (bs, 2H), 2.77 (bs, 1H), 2.54 (bs, 1H), 1.71 (bs, 6H).

^{13}C NMR (100 MHz, $CDCl_3$): δ 147.0, 138.6, 134.3, 123.3, 116.9, 54.4, 48.4, 45.9, 31.8, 27.6.

LRMS (APCI) m/z ($C_{13}H_{16}N_2$): theor. 200.13, exp. 200.9.

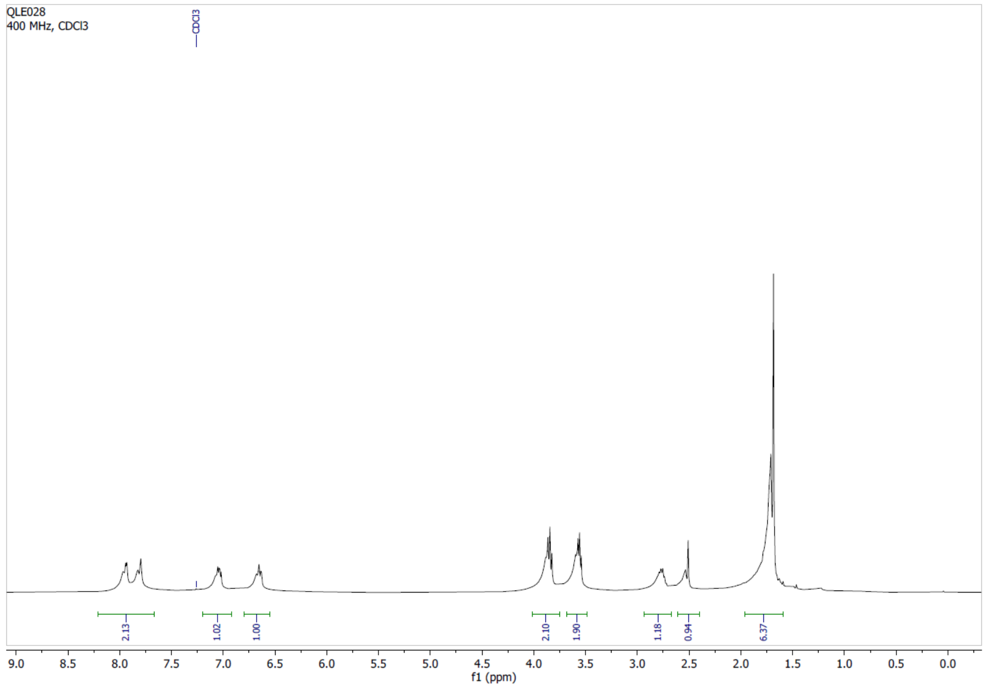

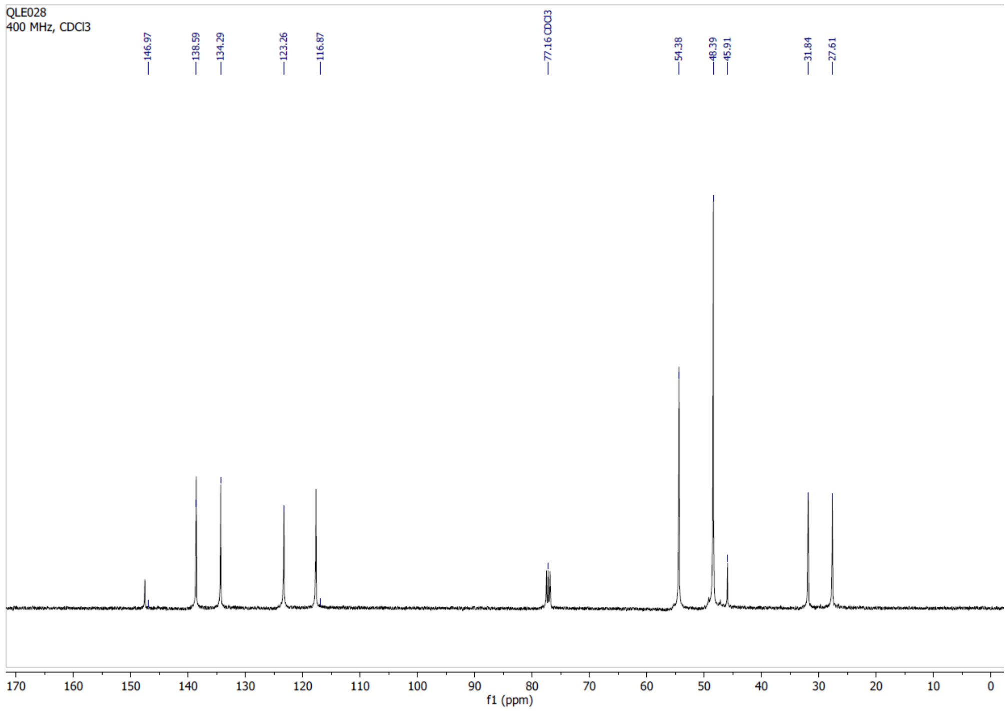

3-(3,3-Difluoroazetidin-1-yl)pyridine (2b)

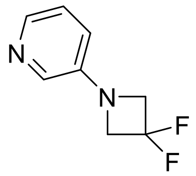

Yellow solid, 100 mg, 78% yield.

^{1}H NMR (400 MHz, $CDCl_3$): δ 8.10 (bs, 1H), 7.89 (bs, 1H), 7.15 (bs, 1H), 6.78 (bs, 1H), 4.28-4.21 (m, 4H).

^{13}C NMR (100 MHz, $CDCl_3$): δ 145.3, 140.5, 135.0, 122.9, 118.9, 115.8 (t, J_{CF} 275 Hz), 63.4 (t, J_{CF} 26 Hz).

LRMS (APCI) m/z ($C_8H_8F_2N_2$): theor. 170.07, exp. 170.7.

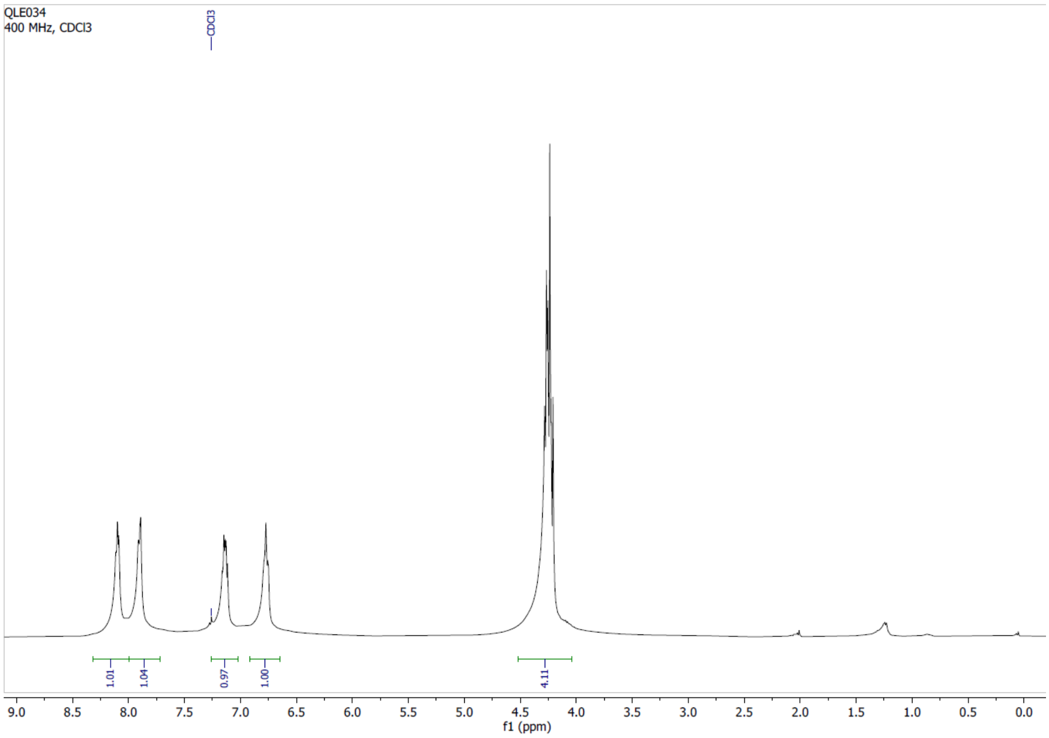

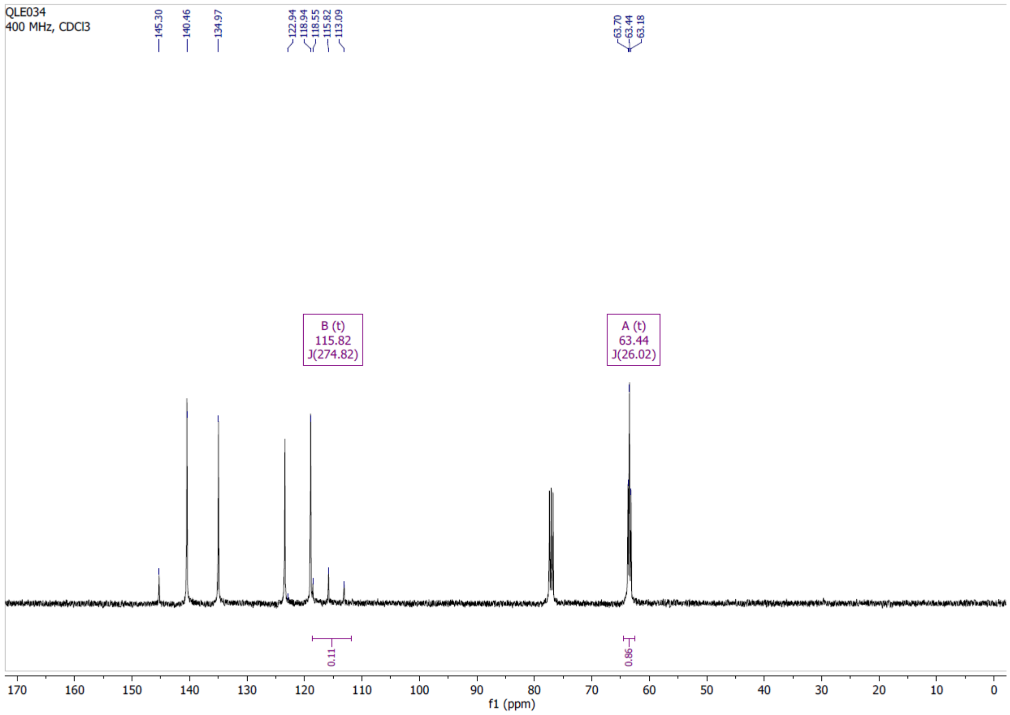

tert-Butyl (1-(pyridin-3-yl)azetidin-3-yl)carbamate (**2c**)

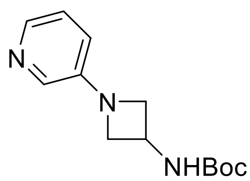

Yellow oil, 43 mg, 23% yield.

^{1}H NMR (400 MHz, $CDCl_3$): δ 7.95 (d, 1H, *J* 4.8 Hz), 7.78 (s, 1H), 7.03 (dd, 1H, *J* 8.2, 4.8 Hz), 6.65 (ddd, 1H, *J* 8.2, 2.9, 1.4 Hz), 5.76 (bs, 1H), 4.62 (bs, 1H), 4.15 (t, 2H, *J* 7.5 Hz), 3.60-3.57 (m, 2H), 1.40 (s, 9H).

^{13}C NMR (100 MHz, $CDCl_3$): δ 155.1, 147.0, 139.1, 134.4, 123.4, 118.3, 79.9, 59.7, 42.0, 28.4.

LRMS (APCI) m/z ($C_{13}H_{19}N_3O_2$): theor. 249.15, exp. 249.7.

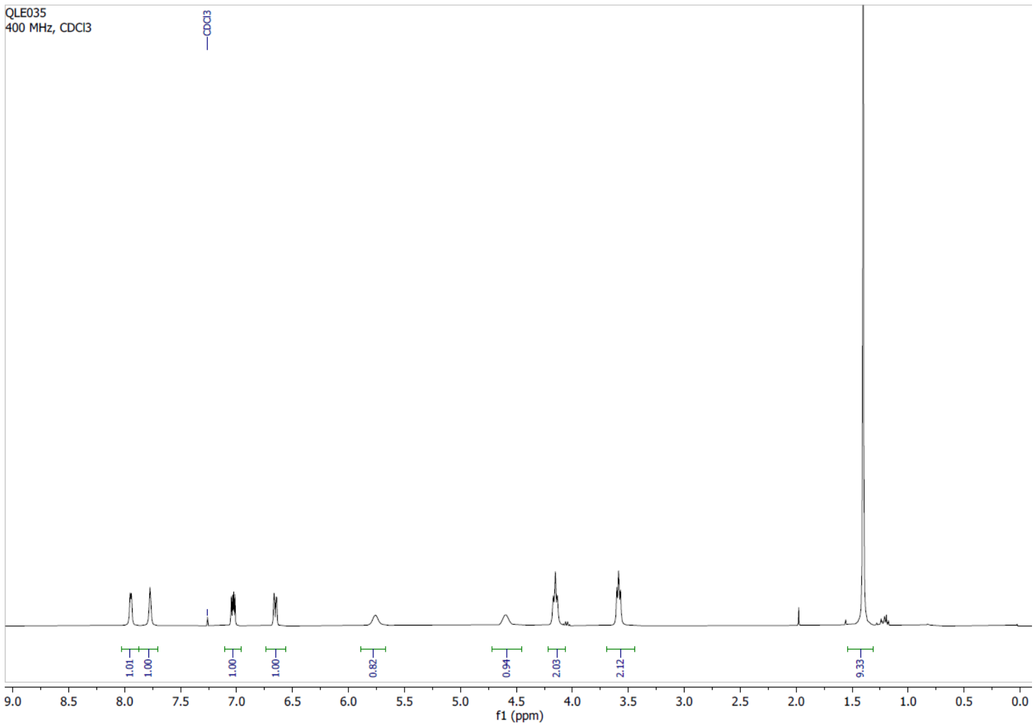

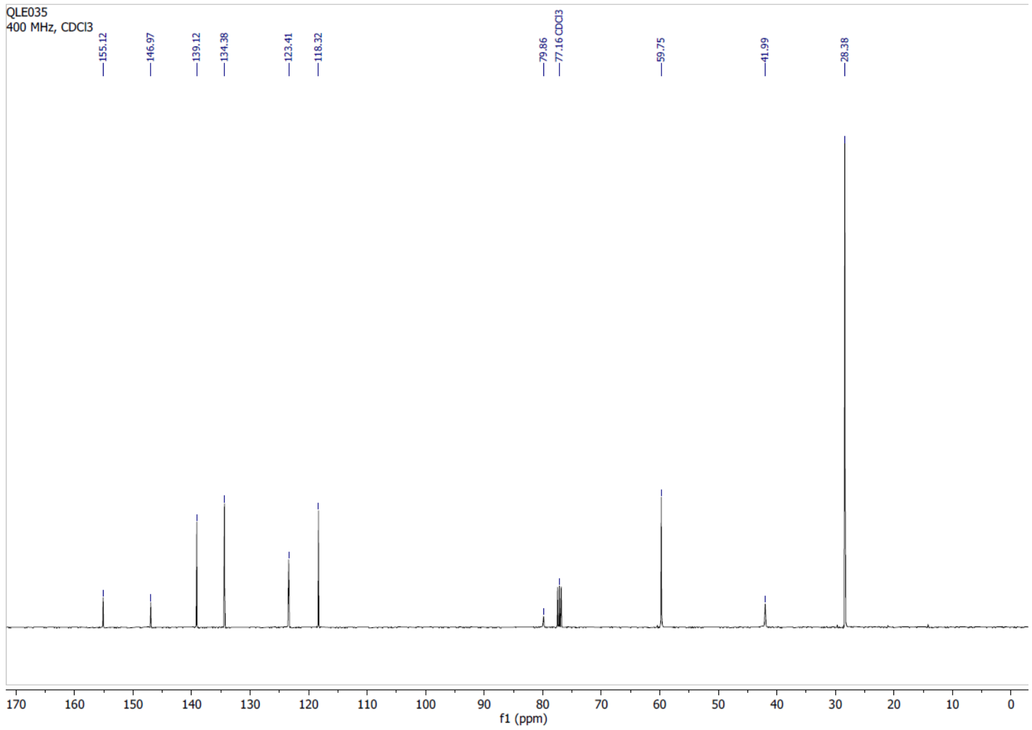

2-(Pyridin-3-yl)-8-oxa-2-azaspiro[4.5]decane (**4c**)

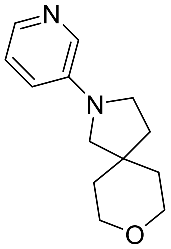

Yellow oil, 38 mg, 23% yield.

^{1}H NMR (400 MHz, $CDCl_3$): δ 7.93 (s, 1H), 7.89 (d, 1H, *J* 4.8 Hz), 7.06 (dd, 1H, *J* 8.4, 4.8 Hz), 6.75 (ddd, 1H, *J* 8.4, 3.0, 1.3 Hz), 3.74-3.60 (m, 4H), 3.33 (t, 2H, *J* 7.0 Hz), 3.16 (s, 2H), 1.90 (t, 2H, *J* 7.0 Hz), 1.60 (t, 4H, *J* 5.4 Hz).

^{13}C NMR (100 MHz, $CDCl_3$): δ 143.7, 137.1, 134.1, 123.6, 117.6, 65.3, 57.7, 45.8, 39.9, 36.3, 35.8.

LRMS (APCI) m/z ($C_{13}H_{18}N_2O$): theor. 218.14, exp. 218.7.

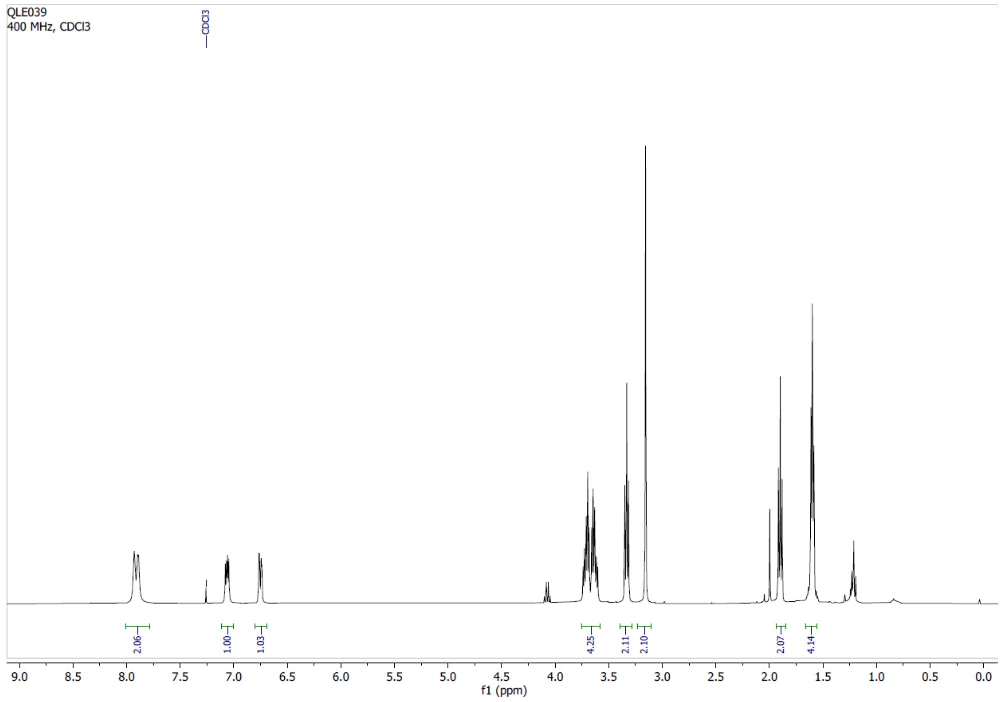

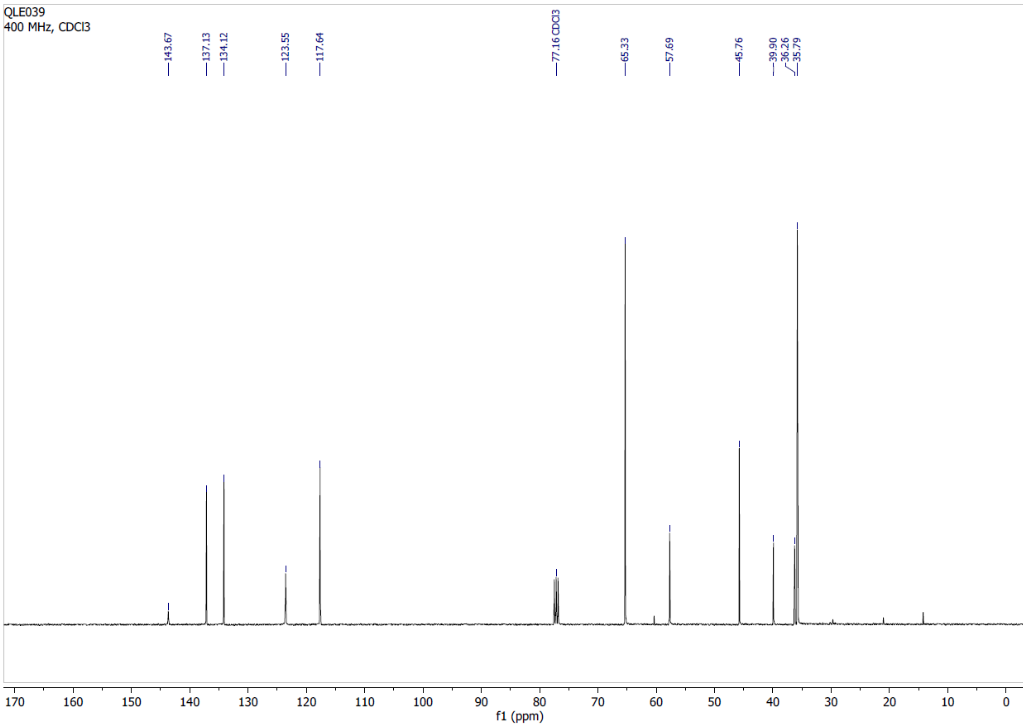

tert-Butyl 6-(pyridin-3-yl)-2,6-diazaspiro[3.4]octane-2-carboxylate (**4d**)

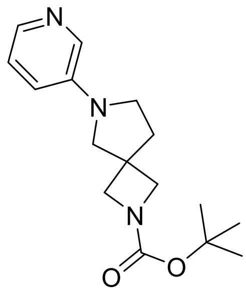

Yellow solid, 140 mg, 65% yield.

^{1}H NMR (400 MHz, $CDCl_3$): δ 7.95 (app d, 2H, *J* 3.2 Hz), 7.11 (dd, 1H, *J* 8.4, 4.6 Hz), 6.75 (dd, 1H, *J* 8.4, 3.0Hz), 3.94-3.87 (m, 4H), 3.46 (s, 2H), 3.37 (t, 2H, *J* 6.8 Hz), 2.21 (t, 2H, *J* 6.8 Hz), 1.44 (s, 9H).

^{13}C NMR (100 MHz, $CDCl_3$): δ 156.4, 143.4, 137.8, 134.3, 123.7, 118.0, 79.9, 59.3 (bs), 57.2, 46.4, 39.9, 36.1, 28.5.

LRMS (APCI) m/z ($C_{16}H_{23}N_3O_2$): theor. 289.18, exp. 289.7.

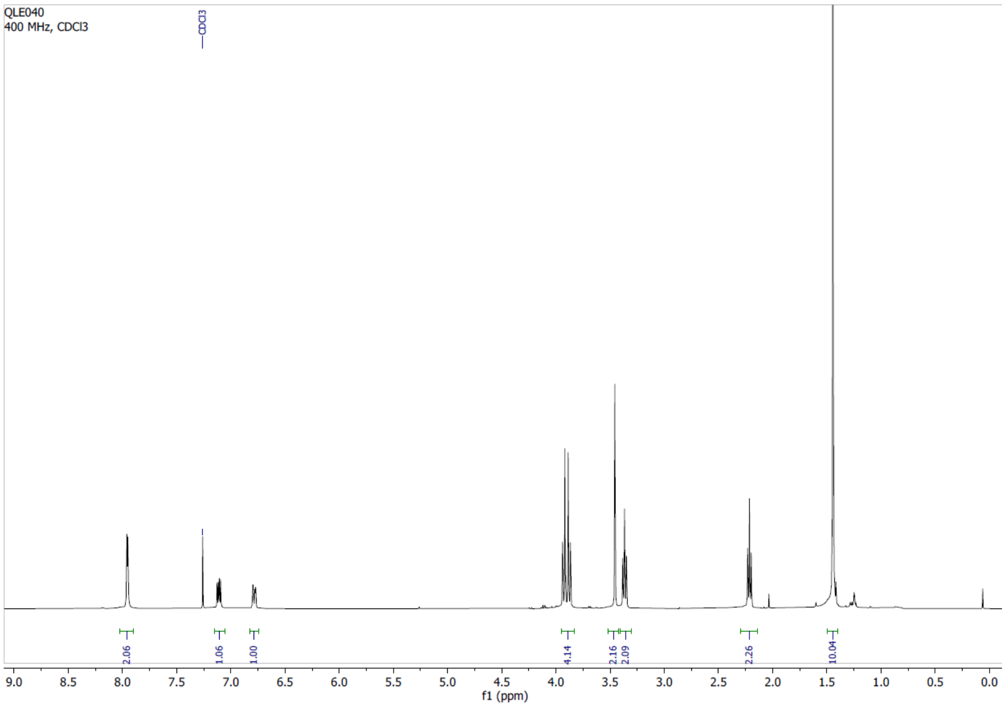

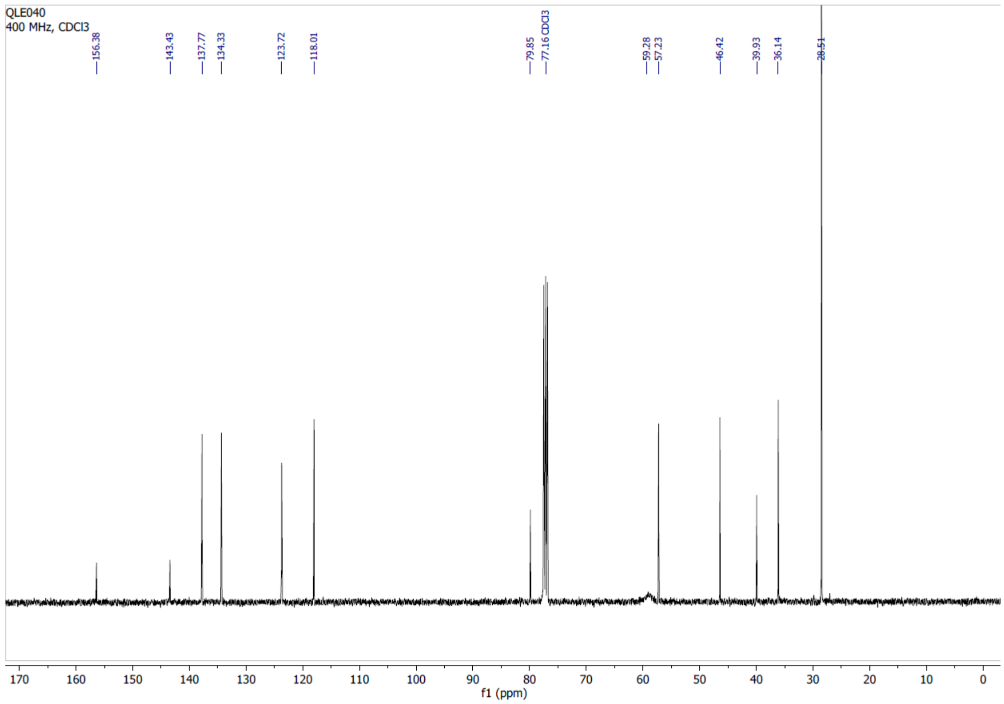

1,1-Difluoro-6-(pyridin-3-yl)-6-azaspiro[2.5]octane (**5a**)

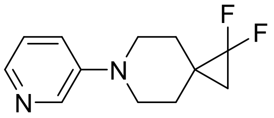

Yellow oil, 25 mg, 15% yield.

^{1}H NMR (400 MHz, $CDCl_3$): δ 8.32 (d, 1H, *J* 2.8 Hz), 8.08 (d, 1H, *J* 4.0 Hz), 7.22-7.13 (m, 2H), 3.32 (ddd, 2H, *J* 12.5, 6.6, 4.0 Hz), 3.30 (ddd, 2H, *J* 12.5, 7.8, 4.0 Hz), 1.84-1.71 (m, 4H), 1.13 (t, 2H, J_F 8.4 Hz).

^{13}C NMR (100 MHz, $CDCl_3$): δ 147.2, 140.7, 139.4, 123.7, 123.3, 115.9 (t, J_{CF} 289 Hz), 48.7, 28.5, 27.0 (t, J_{CF} 10.1 Hz), 21.5 (t, J_{CF} 10.1 Hz).

LRMS (APCI) m/z ($C_{12}H_{14}F_2N_2$): theor. 224.11, exp. 225.0.

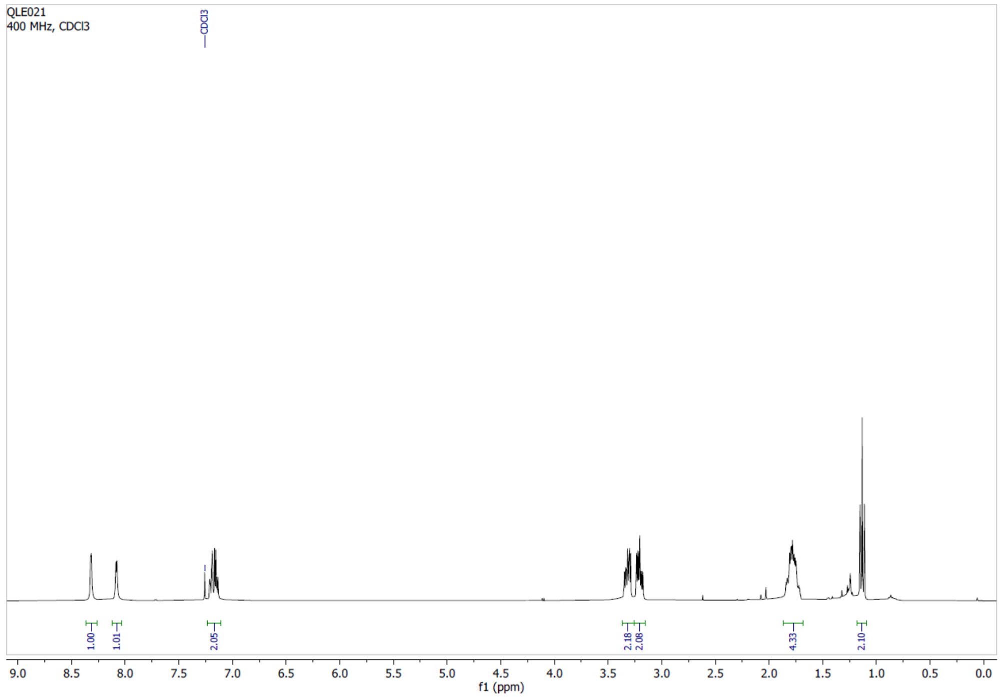

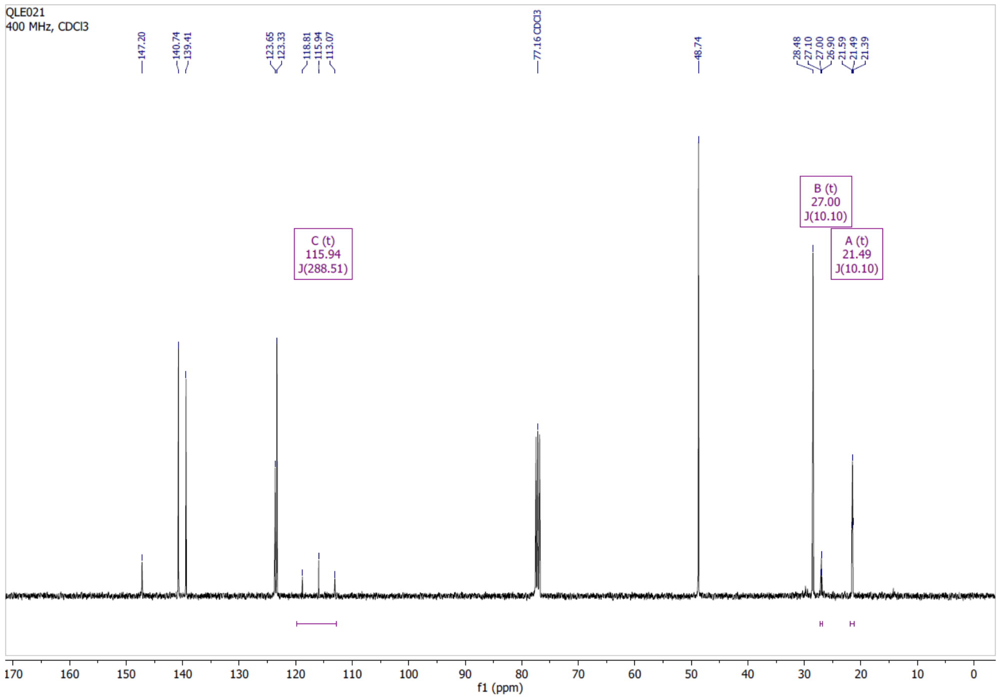

(1S,4S)-5-(Pyridin-3-yl)-2-oxa-5-azabicyclo[2.2.1]heptane (**5b**)

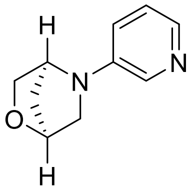

Yellow oil, 29 mg, 22% yield.

^{1}H NMR (400 MHz, $CDCl_3$): δ 7.99 (s, 1H), 7.96 (s, 1H), 7.10 (dd, 1H, *J* 8.4, 4.6 Hz), 6.83 (ddd, 1H *J* 8.4, 3.0, 1.3 Hz), 4.64 (s, 1H), 4.40 (s, 1H), 3.84 (s, 2H), 3.54 (d, 1H, *J* 9.2 Hz), 3.13 (d, 1H, *J* 9.2 Hz), 2.01-1.94 (m, 2H).

^{13}C NMR (100 MHz, $CDCl_3$): δ 143.19, 138.27, 135.45, 123.74, 119.37, 76.22, 71.81, 57.95, 56.99, 36.96.

LRMS (APCI) m/z ($C_{10}H_{12}N_2O$): theor. 176.09, exp. 176.8.

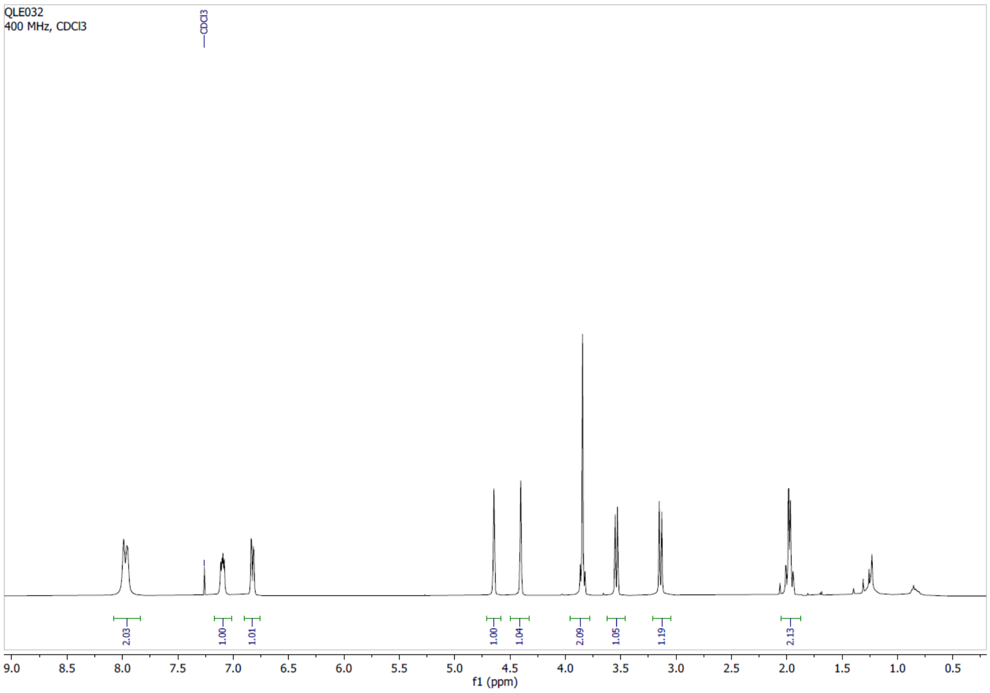

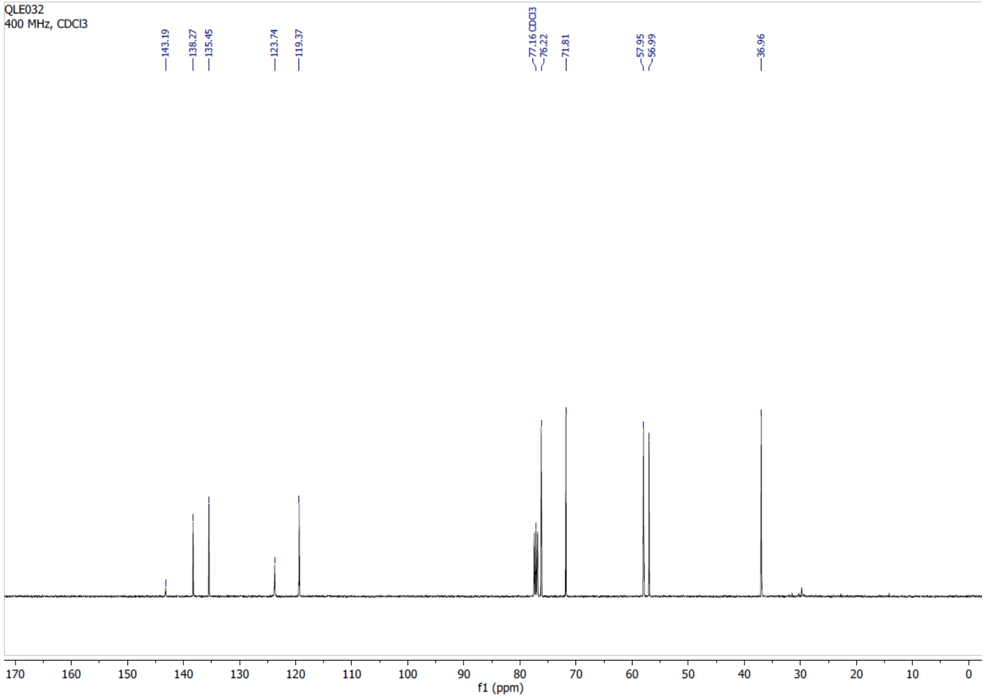

Supplement: File 1 — Experimental part. [file Beilstein_J_Org_Chem-16-982-s001.pdf]
